# Possible sarcopenia and associated factors in community-dwelling older adults with a history of falls in the Republic of Korea: a cross-sectional study

**DOI:** 10.1186/s12877-025-06248-2

**Published:** 2025-07-31

**Authors:** Gwang Suk Kim, Jae Jun Lee, Min Kyung Park, Layoung Kim, Sooyoung Kwon, Eun Ju Park, HaYeJin Yang, SeungBum Yang

**Affiliations:** 1 Mo-Im Kim Nursing Research Institute, College of Nursing, Yonsei University, Seoul, Republic of Korea; 2 College of Nursing · Brain Korea 21 FOUR Project, Yonsei University, Seoul, Republic of Korea; 3 Department of Nursing, Graduate School of Yonsei University, Seoul, Republic of Korea

**Keywords:** Accidental falls, Aged, Early diagnosis, Muscle strength, Risk factors, Sarcopenia

## Abstract

**Background:**

Sarcopenia is a key modifiable risk factor for falls in older adults with a history of falls. However, its diagnosis is a challenge owing to limited resources in primary care or community settings. In 2019, the Asian Working Group for Sarcopenia introduced the concept of ‘possible sarcopenia’ as an easily accessible diagnostic criterion for at-risk individuals. This study aimed to explore factors associated with possible sarcopenia in older adults with a history of falls.

**Methods:**

A cross-sectional study was conducted among 211 older adults aged 65 years and older with at least one fall in the past two years. Possible sarcopenia was identified using a stepwise approach: participants with low calf circumference (< 34 cm for men, < 33 cm for women) underwent further assessments of muscle strength (handgrip strength) and physical performance (5-time chair stand test). Those with low calf circumference along with either low muscle strength (< 28 kg for men, < 18 kg for women) or poor physical performance (≥ 12 s) were classified as having possible sarcopenia. Multiple logistic regression analysis was performed to identify factors associated with possible sarcopenia.

**Results:**

Among the older adults with a history of falls, 27.5% were identified as having possible sarcopenia. Significantly associated factors included older age (≥ 75 years), living alone, unemployment, and depressive symptoms, whereas sex, alcohol consumption, smoking, recurrent falls, and the fear of falling were not significant.

**Conclusions:**

Possible sarcopenia is common in older adults with a history of falls and is significantly associated with advanced age, living alone, unemployment, and depressive symptoms. Therefore, early screening and targeted multifaceted interventions are crucial in mitigating sarcopenia.

**Clinical trial number:**

Not applicable.

**Supplementary Information:**

The online version contains supplementary material available at 10.1186/s12877-025-06248-2.

## Background

Fall is a serious public health concern in older adults [1]. Approximately one in four older adults experiences a fall every year [2], and those who have fallen are twice as likely to fall again than those who have not [3]. Those with a history of falls face an increased risk of fractures, disability, hospitalization, and mortality, placing substantial burdens on caregivers and society [3–5]. Therefore, understanding the characteristics of community-dwelling older adults with a history of falls and addressing modifiable factors are important challenges in promoting healthy aging.

Sarcopenia, characterized by progressive muscle loss, is a key modifiable risk factor for falls [6, 7]. Older adults with sarcopenia had about 2–3 times higher risk of falls than those without sarcopenia [8, 9]. In particular, sarcopenia significantly increases the risk of recurrent falls more than twofold in older adults with a history of falls [10], highlighting a greater concern for this group. Maintaining adequate muscle strength is essential for the daily activities and independence of older adults [7, 11]. Therefore, early diagnosis and management of sarcopenia are crucial, as lifestyle interventions and targeted treatments can help slow or even reverse muscle loss [12] and reduce fall risk in older adults with a history of falls [8–10].

Traditionally, diagnosing sarcopenia requires advanced techniques, such as dual-energy X-ray absorptiometry or bioelectrical impedance analysis [12]. However, these methods require specialised equipment and professionally trained–individuals [13]. Therefore, the diagnosis of sarcopenia is limited to primary care and community settings. To address these limitations, the Asian Working Group for Sarcopenia (AWGS) introduced the concept of ‘possible sarcopenia’, a simplified and easily accessible diagnostic criterion [12]. Possible sarcopenia is defined as reduced muscle strength or physical function [12]. Calf circumference, SARC-F, or SARC-CalF questionnaires are used for case-finding, followed by muscle strength or physical performance assessments if positive [12]. The measurement of possible sarcopenia can facilitate timely intervention strategies to slow or even reverse its progression, and is especially useful in primary care and community settings where resources for advanced muscle-mass measurement are often unavailable [12].

Despite the importance of possible sarcopenia, a research gap on the proportion and associated factors of possible sarcopenia in older adults exists. Previous studies have reported the proportion for possible sarcopenia among older adults ranging from 2.9 to 68.7% [14–21], indicating considerable variation in the reported rates. Several factors, including age, sex, body mass index, and fall history, appear to contribute to the development of possible sarcopenia in older adults, but findings across studies are inconsistent [14–17, 21]. Notably, to the best of our knowledge, no study has focused on older adults with a history of falls, who may be particularly vulnerable to sarcopenia and its effects. Therefore, this study aimed to explore its associated factors among community-dwelling older adults with a history of falls.

## Methods

### Study design

A cross-sectional study was conducted using a home-visit survey.

### Study participants

We included older adults aged 65 years or older who had experienced at least one fall in the past two years, had not relocated during this two-year period, and were living in the Seoul or Gyeonggi regions of the Republic of Korea. Those who could not communicate in Korean or had difficulty in understanding the survey questionnaire were excluded. A total of 211 individuals participated in this study. The final sample size exceeded the sample size of previous studies that examined the factors associated with possible sarcopenia [22–24], indicating sufficient statistical power. Furthermore, given that we performed multiple logistic regression analysis with 10 independent variables and had 58 events of possible sarcopenia, the events per variable was 5.8; this meets the commonly accepted threshold of ≥ 5, ensuring the statistical validity of our regression analysis [25].

### Data collection

Data were collected from December 2022 to July 2023 through home visit surveys. Convenience sampling was used to recruit the target population. Six trained investigators posted recruitment bulletins at senior centres, welfare centres, and churches. Before enrolment, investigators screened participants for eligibility based on predefined inclusion and exclusion criteria. All participants received an information sheet explaining the study objectives, procedures, and estimated completion time before providing informed consent. The investigators visited the participants’ homes and obtained informed consent after explaining the research objectives and study duration and estimated the survey-completion time. All the participants received a gift card worth approximately US$20 as an appreciation token.

### Measurements

#### Possible sarcopenia

Possible sarcopenia was assessed using the AWGS 2019 consensus [12]. According to the guidelines, possible sarcopenia was initially identified through a case-finding step using calf circumference. Investigators measured the maximum circumference of both calves using a nonelastic tape. Participants with calf circumference below the cut-off values (< 34 cm for men, < 33 cm for women) subsequently underwent additional assessment for muscle strength and physical performance. Muscle strength was assessed by measuring the handgrip strength. The participants were asked to squeeze a hand dynamometer (Lavisen KS-301; Lavisen Co. Ltd., Namyangju, Korea) as hard as possible, with each hand being tested twice. The maximum value of four measurements was recorded, and the low muscle strength was defined as < 28 kg for men and < 18 kg for women. Physical performance was measured by the 5-time chair stand test. The participants were asked to sit on a chair with their arms folded in front of their chest, stand up, and sit down five times in a row as quickly as possible without moving their arms. The time taken to complete the task was recorded, with poor physical performance defined as requiring ≥ 12 s. Possible sarcopenia was thus operationally defined as having low calf circumference along with either low muscle strength or poor physical performance.

#### Covariates

Covariates were selected based on previous studies on possible sarcopenia [15–18], which identified age, sex, multimorbidity, living alone, employment status, alcohol consumption, smoking, recurrent falls, fear of falling, and depressive symptoms as relevant factors. These variables reflect a multidimensional set of risk domains—biological, clinical, socioeconomic, behavioural, and psychological—that are known to be associated with muscle weakness and poor physical performance. Multimorbidity was categorized as having two or more chronic conditions. Participants were asked how many falls they had experienced in the past two years and responded with the number of fall events. A fall was defined as an unexpected event in which the participant comes into contact with the ground, floor, or lower level. Two or more falls within the past two years were defined as recurrent falls, whereas only one fall was categorized as a single fall. The participants’ fear of falling was measured with a single-item question of whether they restricted any activities owing to their fear of falling, with response options of *no or yes*. Depressive symptoms were measured using the Korean version of the 15-item geriatric depression scale (GDS-15), and participants with scores ≥ 5 were classified as having depressive symptoms based on a meta-analysis identifying this cut-off as optimal for detecting depression (sensitivity = 0.89, specificity = 0.77) [26].

### Statistical analysis

Descriptive statistics were presented as frequencies and percentages for categorical variables. We conducted the chi-square test to examine differences in covariates between the participants with and without possible sarcopenia. We used the multiple logistic regression analysis to calculate the odds ratio (OR) and 95% confidence interval (95% CI) to determine the associated factors of possible sarcopenia. Model fit was evaluated using the Hosmer-Lemeshow test for goodness-of-fit, and discrimination was assessed with the area under the receiver operating characteristic curve (AUC-ROC). To assess multicollinearity among the independent variables, we computed the variance inflation factor (VIF). A VIF value exceeding 10 was considered indicative of collinearity, with observed VIF values ranging from 1.05 to 1.31. All statistical analyses were performed using SAS (version 9.4; SAS Institute Inc.), and the statistical significance level was set at *p* <.05.

## Results

### Proportion of participants with possible sarcopenia

**Fig. 1:**
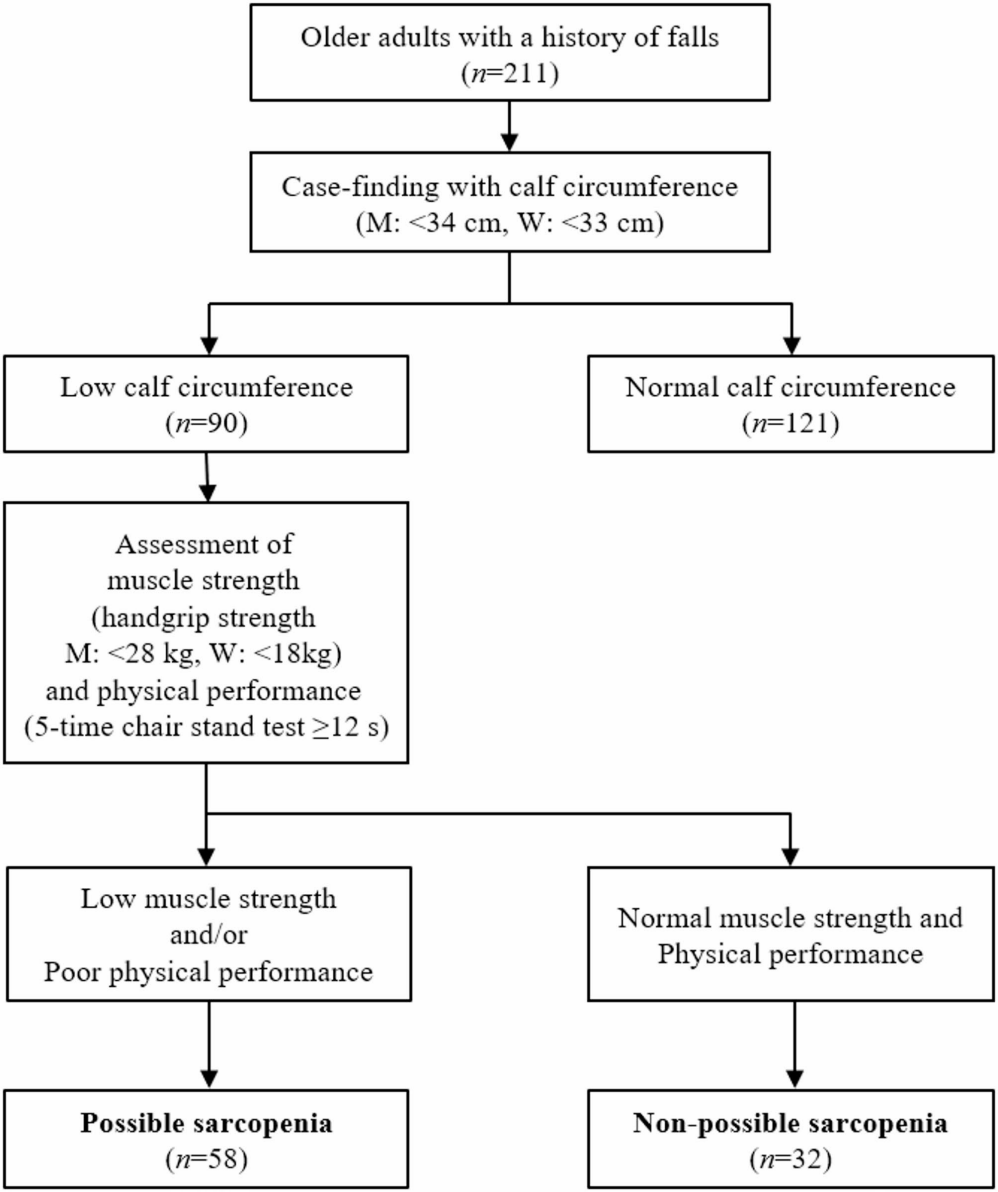
Flow diagram of the screening for possible sarcopenia

As shown in Fig. 1, case-finding for possible sarcopenia was conducted using calf circumference, with 90 of the 211 participants classified as having low calf circumference (M: <34 cm, W: <33 cm). Among them, muscle strength was assessed using handgrip strength (M: <28 kg, W: <18 kg), and physical performance was evaluated using the 5-time chair stand test (≥ 12 s). A total of 58 participants showed low muscle strength and/or poor physical performance. Consequently, 27.5% (*n* = 58/211) of the participants were identified as having possible sarcopenia.

### General characteristics of the study participants

Table 1 shows the proportion and differences in the presence of possible sarcopenia according to the general characteristics of the participants. The mean age of the participants was 74.4 ± 6.5 years, with 93 individuals (44.1%) being aged 75 or more. Of the participants, 46 (21.8%) were men. The proportion of possible sarcopenia was significantly higher in individuals aged 75 years and older (38.7%) than those aged 65–74 years (18.6%) (χ^2^ = 10.5, *p* =.001), whereas there was no difference by sex (χ^2^ = 0.8, *p* =.379) and multimorbidity (χ^2^ = 0.3, *p* =.559). The participants who were living alone had a significantly higher proportion of possible sarcopenia (37.9%) than those who were living with others (23.5%) (χ^2^ = 4.4, *p* =.036). Employed older adults had a significantly lower proportion of possible sarcopenia (13.5%) than unemployed older adults (32.1%) (χ^2^ = 6.8, *p* =.009). Additionally, a significantly higher proportion of possible sarcopenia was observed in older adults with depressive symptoms (41.7%) than those without depressive symptoms (21.9%) (χ^2^ = 8.5, *p* =.004).

**Table 1:** General characteristics of the study participants (*N* = 211)

Variables	Normal (*n* = 153, 72.5%)	Possible sarcopenia (*n* = 58, 27.5%)	x^2^	*P*-value
Age			10.5	0.001
65–74	96 (81.4)	22 (18.6)		
75 or over	57 (61.3)	36 (38.7)		
Sex			0.8	0.379
Men	31 (67.4)	15 (32.6)		
Women	122 (73.9)	43 (26.1)		
Multimorbidity			0.3	0.559
Yes	86 (74.1)	30 (25.9)		
No	67 (70.5)	28 (29.5)		
Living alone			4.4	0.036
Yes	36 (62.1)	22 (37.9)		
No	117 (76.5)	36 (23.5)		
Employment status			6.8	0.009
Yes	45 (86.5)	7 (13.5)		
No	108 (67.9)	51 (32.1)		
Drinking alcohol			0.4	0.527
Yes	38 (76.0)	12 (24.0)		
No	115 (71.4)	46 (28.6)		
Smoking			1.0	0.309
Yes	2 (50.0)	2 (50.0)		
No	151 (72.9)	56 (27.1)		
Recurrent falls			0.1	0.804
Yes	50 (71.4)	20 (28.6)		
No	103 (73.1)	38 (26.9)		
Fear of falling			0.2	0.652
Yes	30 (69.8)	13 (30.2)		
No	123 (73.2)	45 (26.8)		
Depressive symptoms			8.5	0.004
Yes	35 (58.3)	25 (41.7)		
No	118 (78.1)	33 (21.9)		

### Factors associated with possible sarcopenia from multiple logistic regression analysis

Table 2 presents the factors associated with possible sarcopenia identified using the multiple logistic regression analysis. Adults aged 75 or older (OR = 2.38, 95% CI = 1.18–4.78), who were living alone (OR = 2.18, 95% CI = 1.04–4.59), were unemployed (OR = 2.77, 95% CI = 1.06–7.28), and had depressive symptoms (OR = 2.32, 95% CI = 1.13–4.80) had a significantly higher likelihood of possible sarcopenia than those without these factors. Sex, multimorbidity, alcohol consumption, smoking status, recurrent falls, and the fear of falling were not significantly associated with possible sarcopenia. The Hosmer-Lemeshow test indicated good model fit (χ²=10.6, *p* =.226). The AUC was 0.726 (95% CI: 0.650–0.802), indicating acceptable discriminatory power of the logistic regression model (Supplementary File Figure S1).

**Table 2:** Factors associated with possible sarcopenia from multiple logistic regression analysis

Variables	Adjusted OR	95% CI	*P*-value
Age			
65–74	1	-	-
75 or over	2.38	(1.18–4.78)	0.015
Sex			
Men	1	-	-
Women	0.43	(0.18–1.08)	0.071
Multimorbidity			
Yes	0.58	(0.29–1.16)	0.121
No	1	-	-
Living alone			
Yes	2.18	(1.04–4.59)	0.039
No	1	-	-
Employment status			
Yes	1	-	-
No	2.77	(1.06–7.28)	0.038
Drinking alcohol			
Yes	0.68	(0.28–1.65)	0.398
No	1	-	-
Smoking			
Yes	3.01	(0.33–27.43)	0.328
No	1	-	-
Recurrent falls			
Yes	1.05	(0.52–2.11)	0.889
No	1	-	-
Fear of falling			
Yes	0.70	(0.30–1.65)	0.420
No	1	-	-
Depressive symptoms			
Yes	2.32	(1.13–4.80)	0.022
No	1	-	-

*Abbreviations*: *OR* Odds ratio, *CI* Confidence interval

## Discussion

To the best of our knowledge, this is the first study to investigate the factors associated with possible sarcopenia in older adults with a history of falls. We found that 27.5% of the participants exhibited possible sarcopenia, underscoring the considerable proportion of this condition. Several factors, including age, living alone, employment status, and depressive symptoms, were significantly associated with possible sarcopenia. However, due to the cross-sectional design of our study, we cannot establish causality, and longitudinal studies are required to further clarify these relationships. Our findings provide foundational information on possible sarcopenia in older adults with a history of falls and contribute to the development of targeted interventions.

This study found that the proportion of participants with possible sarcopenia was slightly higher than that reported by previous studies using the same measurement methods to diagnose possible sarcopenia. Among 2,213 Korean older adults with a mean age of 75.9 years, 479 (22.6%) were identified as having possible sarcopenia [18]. Similarly, in a group of 349 older Japanese adults with a mean age of 78.0 years who visited a frailty clinic at a geriatric hospital, 86 (24.6%) were classified as having possible sarcopenia [27]. Our focus on older adults with a history of falls, a population particularly vulnerable to sarcopenia owing to decreased mobility and physical activity [28], may explain the relatively higher proportion of participants identified with possible sarcopenia than prior studies among older adult population which did not consider the history of a fall [18, 27]. This finding highlights the need for early identification and targeted interventions to prevent further functional decline in this vulnerable population.

However, the identification rate of possible sarcopenia varies considerably across studies due to differences in measurement approaches [16, 18, 29]. For example, Kim and Won [18] showed that the prevalence ranged from 3.1–24.6%, depending on tool combinations [18]. Such variability poses challenges for comparing and integrating findings from previous research, underscoring need to standardise measurement methods [30, 31]. A previous study indicated that calf circumference is a better tool for screening sarcopenia than the SARC-F or SARC-CalF [32]. Another study demonstrated that handgrip strength is a more useful biomarker for predicting possible sarcopenia than the 5-time chair stand test, and that combining the two tests provides higher diagnostic accuracy than using either test alone, recommending the use of both; however, if resources are limited, prioritising the handgrip dynamometer is advised [27]. Our study derived case-finding using calf circumference and performed additional assessments using handgrip strength and the 5-time chair stand test, making it an appropriate method for measuring possible sarcopenia. However, research on the optimal measurement methods for predicting sarcopenia remains limited, highlighting the need for further investigation to determine the best combination of case-findings and assessment tools based on predictive accuracy. In the meantime, healthcare providers may choose screening tools based on patient characteristics and care settings. Calf circumference may be preferred in home visits or primary care due to its simplicity, whereas SARC-F or SARC-CalF could be used where physical measurements are less feasible. Ideally, both the 5-time chair stand test and handgrip strength should be used together for greater accuracy. When only one assessment is feasible, handgrip strength may be prioritized, given its stronger predictive validity compared to the 5-time chair stand test. This stepwise and flexible screening approach may facilitate early detection and targeted intervention in older adults who are at risk, especially in resource-limited clinical and community settings.

Additionally, the simplified definition of possible sarcopenia in the AWGS 2019 guidelines has caused confusion in diagnosis. According to AWGS guidelines, diagnosing possible sarcopenia requires a two-step process: initial case-finding that identifies individuals at risk, followed by a secondary assessment of muscle strength and/or physical performance. However, the AWGS 2019 definition describes possible sarcopenia simply as ‘low muscle strength with or without reduced physical performance’, without explicitly mentioning the need for the case-finding [12]. This ambiguity has led to confusion regarding the necessity of prior case-finding, and several studies actually omitted this initial step [33, 34]. Therefore, we recommend that the AWGS 2019 incorporate case-finding related content into the formal definition of possible sarcopenia.

Calf circumference, handgrip strength, and the 5-time chair stand test, as used in this study, offer practical advantages in that they require no specialized equipment or advanced training. These features make them suitable for implementation in diverse clinical and community-based settings, including fall clinics, primary care visits, and community health outreach programs [12]. However, their effective integration into routine clinical practice requires organizational support and policy-level efforts, in order to incorporate these tools as standard components of existing health assessment programs [12]. For instance, routinely including calf circumference and handgrip strength measurements in older adults’ regular health screenings, combined with clearly defined clinical processes that link identified high-risk individuals to tailored strength training and nutritional intervention programs, could be a practical approach in clinical settings. This approach aligns with the World Health Organization’s integrated care for older people guidelines, which emphasize systematic screening, structured referral pathways, and sustained support through community resources and personalized care plans [35]. Additionally, the integrated care of older patients with frailty in primary care study conducted in Korea demonstrated that integrating structured geriatric assessments within primary care, supported by health coaching and periodic follow-up visits, effectively facilitates early detection and intervention for frailty and related functional impairments [36]. To operationalize this approach, standardized protocols should be developed and disseminated, covering assessment procedures, interpretation of findings, and subsequent follow-up actions. Targeted training for frontline healthcare providers and community health workers is also essential [7], with an emphasis on recognizing functional decline, conducting basic assessments reliably, and applying clear referral algorithms. In home-visit settings, these protocols could enable healthcare providers to identify at-risk individuals during routine visits and promptly connect them to appropriate community-based interventions and services [35]. Such practical and structured efforts may help in early detection and effective management of possible sarcopenia, potentially reducing fall risk among older adults.

Age was a significant factor associated with possible sarcopenia, with individuals aged 75 years and older showing a higher proportion than those aged 65–74 years. This finding is consistent with previous studies that demonstrated a positive association between age and possible sarcopenia [14, 15, 17]. As individuals age, changes in their skeletal muscle structure and function lead to muscle mass decline [11]. Consequently, the proportion of sarcopenia is expected to increase with age, raising concern, particularly for older adults aged 75 and over who have experienced falls. It is also important to note that possible sarcopenia was prevalent even among participants aged 65–74 (18.6%), suggesting the need for addressing sarcopenia in this comparatively younger age group as well.

Our findings revealed that older adults living alone had a higher likelihood of possible sarcopenia than those living with others, whereas those who were employed had a lower likelihood than their unemployed counterparts. Previous studies have suggested that older adults living alone often experience social isolation and lack of support, potentially associated with reduced physical activity and poor nutritional status, which might contribute to muscle loss [37–39]. Employment status was associated with a lower likelihood of possible sarcopenia. Previous research indicates that employment could be linked to better opportunities for physical activity and nutritional status [40, 41], which might be beneficial for maintaining muscle function [12]. Employment is also associated with a higher socioeconomic status, which can act as a protective factor against sarcopenia by providing benefits such as better access to healthcare [42]. Furthermore, those who already experience possible sarcopenia have physical and functional limitations such as muscle weakness and reduced mobility, which could result in difficulty performing job tasks or even lead to early retirement [43]. Thus, while employment may be beneficial in preventing sarcopenia, those with sarcopenia may struggle to remain employed, creating a mutually influential relationship between sarcopenia and employment. Given that older adults living alone and those who are unemployed are already vulnerable populations with an increased risk of possible sarcopenia, incorporating social and economic factors into sarcopenia-prevention strategies is essential. Comprehensive interventions that simultaneously target muscle health and social vulnerabilities can help mitigate functional decline and improve overall well-being.

This study found depressive symptoms as a key factor associated with possible sarcopenia, in line with previous studies [44–46]. A possible explanation for this association is shared lifestyle factors, such as physical inactivity and poor nutrition. Previous studies suggest that older adults with depressive symptoms might engage less frequently in physical activity, potentially contributing to muscle wasting and the development or progression of sarcopenia [46]. Additionally, older adults with depression have reduced appetite, leading to insufficient intake of essential nutrients, such as protein and vitamin D, which subsequently contributes to muscle weakness and loss [47]. The findings of this study underscore the importance of addressing mental health issues, particularly depressive symptoms, for the management of sarcopenia in older adults with a history of falls. However, due to the cross-sectional design of our study, we cannot establish causality. Future research should focus on longitudinal studies to explore the long-term impact of mental health interventions on sarcopenia outcomes, as well as the mechanisms by which depressive symptoms influence muscle health.

Unlike previous studies [48–50], we did not find significant associations between sarcopenia and other factors such as sex, alcohol consumption, smoking status, recurrent falls, or fear of falling. One possible explanation for these unexpected results may be the specific characteristics of the participants, who consisted of older adults with a history of at least one fall, making them a particularly vulnerable and frail group [51]. This higher baseline level of frailty may have contributed to the lack of statistical significance for these variables, as the participants may have already been at an elevated risk of sarcopenia and its associated complications, regardless of these additional factors. In other words, the homogeneously high vulnerability to sarcopenia within this population may have diminished the relative impact of these individual risk factors compared to studies involving more general populations of older adults [47–49]. Moreover, only 23.7% of participants reported alcohol consumption and 1.9% reported smoking, suggesting limited statistical power to detect associations for these variables. Future studies may benefit from stratifying participants according to their fall history to further explore these associations.

This study has several limitations. First, the cross-sectional design prevented us from establishing causal relationships between the identified factors and sarcopenia. Additionally, as we recruited older adults with a history of falls and assessed the presence of possible sarcopenia, it remains unclear whether falls led to possible sarcopenia or whether possible sarcopenia contributed to prior falls. Individuals with sarcopenia may have impaired muscle strength and physical function that predispose them to falls, thereby raising the possibility of reverse causality. This limitation should be carefully considered when interpreting the findings, and longitudinal studies are required to clarify these associations. Second, several covariates, including fall history, alcohol consumption, and smoking, were assessed through self-reported questionnaires, which may be subject to recall bias and social desirability bias. For instance, older adults may underreport alcohol consumption or smoking due to social stigma, and their recall of fall incidents over the past two years may be incomplete or inaccurate. Furthermore, depressive symptoms were assessed using the GDS, a screening tool rather than a diagnostic instrument for clinical depressive symptoms, potentially leading to measurement inaccuracies. Additionally, the fear of falling was assessed using a single-item question, which may not have fully captured the multifaceted nature of this construct. As such, multi-item validated scales may offer greater sensitivity in detecting its association with possible sarcopenia. Third, we adjusted for several variables to minimize bias; however, some residual confounders, such as body mass index, physical activity level, and nutritional status, might still remain. These factors may also influence the risk of possible sarcopenia, and future research should include these variables to further clarify their impact. Finally, our study employed convenience sampling from limited geographic regions (Seoul and Gyeonggi Province), which may introduce selection bias and limit the generalisability of the findings to a broader population of older adults with a history of falls. Therefore, these results may not fully represent older adults with a history of falls from different community settings or demographic groups, and future studies should consider more representative sampling approaches to enhance external validity.

This study has several strengths. First, we applied standardized diagnostic criterion based on the AWGS 2019 guidelines, ensuring the reliability of possible sarcopenia classification. Second, unlike previous studies that focused on the general older population, our study specifically examined older adults with a history of falls, a population that is particularly vulnerable to sarcopenia but has not been extensively studied. Lastly, we utilized simple and practical screening tools, such as calf circumference and handgrip strength, which can be easily implemented in resource-limited environments.

## Conclusions

In conclusion, this study demonstrated that possible sarcopenia is commonly observed among older adults with a history of falls and is significantly associated with advanced age, living alone, unemployment, and depressive symptoms. These findings underscore the importance of multidimensional interventions, such as physical activity programs, mental health support, and social engagement initiatives, to address not only the physical but also the social and psychological determinants of sarcopenia. Integrating sarcopenia screening into routine geriatric assessments may help to identify at-risk individuals early and facilitate timely interventions. Early screening and targeted prevention efforts could help reduce the burden of sarcopenia and associated functional decline in older adults with a history of falls. Future longitudinal studies are needed to further explore the long-term impact of these factors and better understand the causal relationships involved.

## Supplementary Information

Supplementary Material 1.

## Data Availability

The datasets are not publicly available due to ethical restrictions and the absence of participants’ consent for data sharing. For inquiries regarding the data, please contact the corresponding author, Dr. Jae Jun Lee (email: ljw186111@yuhs.ac).

## Abbreviations

AWGS: Asian Working Group for Sarcopenia
GDS-15: Geriatric depression scale-15
OR: Odds ratio
CI: Confidence interval
VIF: Variance inflation factor
