# Supplementary material for: Possible sarcopenia and associated factors in community-dwelling older adults with a history of falls in the Republic of Korea: a cross-sectional study
Source: BMC Geriatr. 2025 Jul 31;25:575. doi: 10.1186/s12877-025-06248-2 (PMC12315269; doi:10.1186/s12877-025-06248-2)
Supplement: Supplementary file 1 — Supplementary Material 1. [file 12877_2025_6248_MOESM1_ESM.docx]

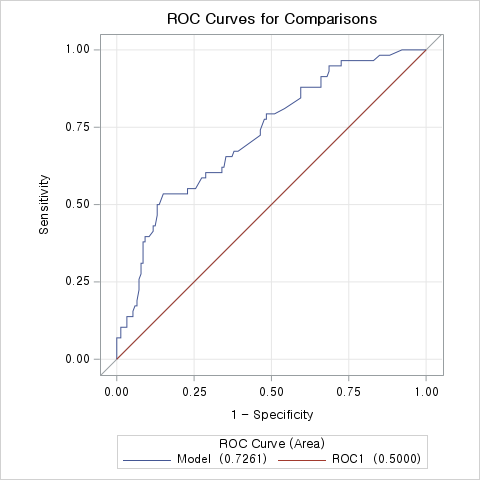


**Supplementary Figure S1**. Receiver operating characteristic (ROC) curves for logistic regression model of possible sarcopenia
